# Employing Genetic Markers to Improve Diagnosis of Thyroid Tumor Fine Needle Biopsy 

**DOI:** 10.2174/138920211798120781

**Published:** 2011-12

**Authors:** Janete M Cerutti

**Affiliations:** Genetic Bases of Thyroid Tumor Laboratory, Division of Genetics, Department of Morphology and Genetics and Division of Endocrinology, Department of Medicine, Federal University of São Paulo, SP, Brazil

**Keywords:** Follicular thyroid carcinoma, follicular thyroid adenoma, indeterminate nodule, FNA, molecular markers, ITM1, C1orf24.

## Abstract

Fine-Needle Aspiration (FNA) is the most widely used and cost-effective preoperative test for the initial evaluation of a thyroid nodule, although it has limited diagnostic accuracy for several types of tumors. Patients will often receive cytological report of indeterminate cytology and are referred to surgery for a more accurate diagnosis. An improved test would help physicians rapidly focus treatment on true malignancies and avoid some unnecessary treatment of benign tumors. This review will discuss current molecular markers that may improve thyroid nodule diagnosis.

## INTRODUCTION

Thyroid nodules are commonly found in clinical practice. An estimated 4-7% of the adult population develop a clinically detectable thyroid nodule during their lifetime and this prevalence increases with age [1]. The advent of thyroid ultrasound, however, allows for an increasing number of nodules to be diagnosed. It is now recognized that nodules are detected at a subclinical level and are present in an estimated 67% of the general population [2,3]. The detection of a subclinical thyroid nodule demands a careful preoperative evaluation, since it may represent a thyroid malignancy. 

Currently, serological tests and modern imaging techniques are employed for the clinical evaluation of a thyroid nodule. It is generally regarded that fine needle aspiration (FNA) cytology is the best non-surgical diagnostic tool for distinguishing a benign from a malignant thyroid nodule. Although FNA is the gold standard test for the preoperative assessment of a thyroid nodule, its accuracy varies greatly with histological subtype. Diagnosis is accurate for papillary thyroid carcinoma (PTC) in approximately 90-100% of FNA specimens. However, FNA accuracy is much lower for nodular hyperplasia (HN), follicular adenoma (FTA), Hürthle cell adenoma (HCA), follicular carcinoma (FTC), Hürthle cell carcinoma (HCC) and the follicular variant of papillary thyroid carcinoma (FVPTC) [4,5]. Notably, a high percentage of nodules classified as indeterminate were subsequently found to be FVPTC [6,7]. 

About 30% of all FNAs will be diagnosed as indeterminate. Only 10-25% of all indeterminate lesions on FNA prove to be malignant, the majority of patients are unnecessarily subjected to resource-intensive surgery and its potential complications [3,8,9].

To improve the diagnostic accuracy, it has been suggested that nodules should be stratified according risk of malignancy. The National Cancer Institute (NCI) recently sponsored a conference to review the State of Science for the use of FNA in the management of thyroid nodule [10]. Among the topics, the experts discussed the diagnostic terminology and morphological criteria for the cytological diagnosis of thyroid lesions. The committee has proposed a six-category scheme, according with the predicted probability of malignancy: (1) benign, (2) follicular lesion of undetermined significance (FLUS), (3) follicular neoplasm (e.g., suspicious for follicular or Hürthle neoplasm), (4) suspicious for malignancy, (5) malignant, and (6) non-diagnostic [11-13]. According to this scheme, the indeterminate category encompasses three subcategories, i.e. 2, 3 and 4. 

Although FNA is an important technique for the triage of thyroid nodules and this system represents a major step forward to the standardization of the thyroid FNA report, there is still a large ‘gray zone’ in FNA-cytology. Therefore, it is clinically important to have molecular markers that, in conjunction with FNA, can identify the subset of patients who have a malignant nodule. 

## *PAX8/PPARγ* REARRANGEMENTS AND *RAS* POINT MUTATIONS AS A DIAGNOSTIC TOOL

In 2000, Kroll *et al*. reported an intrachromosomal translocation involving chromosomal regions 2q13 and 3p25 in human tumors arising from thyroid follicular epithelial cells. This recurrent translocation was reported in five FTCs but was not detected in any benign thyroid lesions or PTC [14]. The authors suggested that *PAX8-PPARγ* may aid in the differential diagnosis of FTC and benign thyroid lesions [14]. 

Further analysis demonstrated that this rearrangement was found in up to 55% of FTAs [15,16]. Additionally, the overall prevalence of *PAX8-PPARγ* rearrangement in FTC and its variant HCC is lower than the initially reported (for details, see review [17]). 

The mechanisms by which *PAX8-PPARγ* may act are not fully understood. Interestingly, a new rearrangement in which a sequence of *PPARγ* is fused to *CREB3L2* gene at chromosome 7q34 (*CREB3L2- PPARγ*) was reported in a FTC [18]*. *Since *PPARγ* was found associated with both rearrangements, it has been suggested that the disruption of this gene may contribute to the pathogenesis of the disease. Several studies demonstrated that the fusion protein increases cell growth, cell viability and induces anchorage independent growth while decreases apoptosis *in vitro*. There is no evidence, however, that this protein affect tumorigenesis *in vivo *(for details see review [19]). Therefore, it is still not clear whether additional events are needed to promote tumorigenesis. 

Few studies have investigated whether activating mutation in *RAS* genes, previously associated with pathogenesis of follicular tumors [20-22], may cooperate with *PAX8/PPARγ* rearrangement in the pathogenesis of FTC. 

Based on combined analysis of mutation profiling (*PAX8/PPARγ* and* RAS*) with HBME1 and Galectin 3 (LGALS3) expression*, *it was suggested that conventional FTC develop through a distinct and non-overlapping pathway: one involving* RAS *and another involving* PAX8/PPARGγ*rearrangement [23]. FTCs with *PAX8/PPARGγ *rearrangement were found to express LGALS3 while FTCs with *RAS* mutation were found to express HBME1. Additionally, the authors observed difference in the clinical-pathological features in the two groups. Tumors harboring PAX8-PPARγ rearrangement tend to present at a younger age, be smaller in size, have a solid/nested growth pattern and, more frequently reveal vascular invasion. 

Given that the *PAX8-PPARγ *rearrangement was found in a higher proportion of FTC (30%) and in one case of FTA with a thick capsule and immunohistochemical profile characteristic of thyroid cancer, the authors suggested it may represent a pre-invasive (*in situ*) FTC in which the invasion was overlooked by the pathologist. In view of the fact that *RAS* mutations were found in similar proportion of FTA (48%) and FTC (52%), the authors also suggested that FTC harboring *RAS* mutations developed through a benign FTA stage and therefore, it may be precursor lesions for *RAS*-positive FTC [23]. It is still not clear yet whether nodules with *RAS* mutations have malignant potential and should be surgically removed. Because *RAS* mutation was also associated with worse prognosis and progression to undifferentiated thyroid carcinoma, some groups claim that surgical removal of *RAS*-positive FTA may be justifiable to prevent tumor progression [24]. 

Intriguingly,* PAX8-PPARγ* and *RAS* mutations were rarely found in HCA and HCC, corroborating with previous analysis which suggested that Hürthle tumors may develop through a distinct pathways [23].

## CAN SOMATIC MUTATIONS ALONG THE RET/RAS/RAF PATHWAY IMPROVE DIAGNOSTIC ACCURACY OF NODULES CLASSIFIED AS INDETERMINATE ON FNA? 

Mutations of genes coding for effectors along the MAPK pathway (RET/PTC, RAS and BRAF) have been described in about 70% of PTC and are mutually exclusive [25]. Although diagnosis of PTC is correct in most of FNA specimens, several studies have investigated whether the use of BRAF V600E or RET/PTC could improve the accuracy of thyroid FNA biopsy [26-31]. Although BRAF or RET/PTC are highly specific for the diagnosis of PTC, they are of limited value in the preoperative diagnosis on those nodules with an indeterminate FNA cytology. 

Recently, few studies assessed feasibility and significance of the use of a panel of tumor-specific mutations in the preoperative diagnosis of a thyroid nodule. The studies, discussed bellow, are summarized in Table **1**.

Nikiforov *et al*., reported that the combination of *BRAF*, *RAS*, *RET/PTC* and *PAX8/PPARγ* mutations improved the accuracy of cytological diagnosis, particularly samples with indeterminate cytology. The detection of any mutation was highly predictive of malignancy; the specificity approached to 100% [24]. Although the authors declared that the test improved the sensitivity, in comparison with cytology alone, the sensitivity was lower (71%) in nodules commonly classified as indeterminate. Additionally, when the cytology samples from indeterminate were reviewed again and assigned to FLUS, follicular neoplasm or suspicious for malignancy subcategories, the sensitivity was 100%, 75% and 60% respectively. Worth mentioning that although *RAS* and *PAX8/PPARγ* were highly specific for malignancy, these mutations have been previously found in FTA and other benign lesions (for details see [17]). 

Cantara *et al*., have also evaluated the diagnostic utility of *BRAF*, *RAS,* *RET/PTC, TRK *and *PAX8/PPARγ *mutations*. *The authors found that this panel of tumor-associated mutations correctly identified cancer in 78.2%, while cytology identified 58.9% of the thyroid cancers. In the group of indeterminate nodules (*n*=41), molecular markers predicted cancer in 6 out of 7 samples. In the group of suspicious for cancer (*n*=54), the panel of markers predicted cancer in 37 samples while missed the diagnosis in 9 samples. All predicted cancer proved to be PTC at final histology (Table **1**). Their results demonstrated that molecular analysis on FNA samples is feasible and that, when including the analysis of a complete panel of oncogenes, increase the diagnostic accuracy of conventional cytology [32].

Mose *et al*., sought to determine the clinical utility of testing *BRAF*, *RAS* *RET/PTC* and *TRK* mutations in thyroid FNA biopsy [33]. Molecular testing for a panel of mutations in thyroid FNA biopsies with indeterminate cytology revealed a sensitivity of 27%, specificity of 95%, PPV of 66% and NPV of 78%. Their results pointed out that this panel of markers missed the diagnosis of cancer in 21 out of 29 samples; all proved to be FTC, HCC and FVPTC at final histology. When considered FNA biopsies that were indeterminate (n=110) and suspicious for malignancy (*n*= 27), sensitivity of 12%, specificity of 98%, PPV of 38% and NPV of 65% (Table **1**). 

Ohori *et al.*, investigated the *BRAF, RAS, RET/PTC* and *PAX8/PPARγ* mutational status of nodules classified as FLUS. The main focus was to determine whether molecular markers could help to better refine FLUS category (with an estimated probability of malignancy between 5 and 10%). Although the molecular test had a high specificity, the sensitivity was quite low (60%). Molecular markers were negative in seven out of 19 cases of PTC. The authors suggested that, although not all PTC were detected by the panel of common molecular markers, a positive molecular test helped to refine the FLUS case into high-risk and low-risk categories [34]. 

A caveat concerning the use of this panel of tumor-associated markers is that *BRAF* mutation is most commonly associated with classical variant of PTC [35,36] and a high percentage of nodules classified into indeterminate category are FVPTC. Moreover, it has been reported that mutations in the tissue samples were not found in cytological material from same patient [32]. It is still unclear whether the false negative results was due to the fact that the FNA biopsy was not representative of the tumor cells or there has been a contamination with DNA or RNA isolated from non-malignant, stromal, inflammatory or blood cells. Finally, searching for *RET/PTC* rearrangement was possible in about 50% of samples due to low quantity of material or bad quality of RNA [32]. Therefore, not only proper collection of samples is a mandatory step but also a reasonably amount of starting material is needed to isolate both DNA and RNA. 

While this diagnostic approach may have lower sensitivity for indeterminate nodules, the preoperative detection of these mutations, particularly for *BRAF*, may help to identify patients at high risk of recurrence and death [35-39]. Beyond the impact in patient management it will probably have an impact in therapeutics [40-42]. 

## MOLECULAR CLASSIFICATION USING EXPRESSION PROFILING

A number of candidate biomarkers have been identified last years. Among the candidate markers that are thought to discriminate the benign from the malignant thyroid carcinoma is LGALS3 (Galectin-3). LGALS3 was initially described as a potential marker that could discriminate benign from well differentiated thyroid carcinomas with high sensitivity and specificity [43,44]. Although some studies confirmed the initial findings, others have found LGALS3 expression in normal thyroid and benign lesions. Additionally, a high percentage of FTC, HCC and FVPTC are negative for LGALS3. It has been suggested that the reasons for this discrepancy might be related to the difference in technical procedures and antibody clone used [7] (for details see [17]). 

Taking into consideration the technical problems, a prospective multicenter study was conducted to test the utility of LGALS3 for testing nodules classified as indeterminate on FNA-cytology [7]. Although LGALS3 had a specificity of 93%, the overall sensitivity of the test was 78%. Although the authors declared that 71% of unnecessary thyroid surgical procedures could be avoided, 29 cancers (22%) would be missed. Considering the implications of false*-*negative findings, the use of LGALS3 alone in clinical practice should be interpreted cautiously. 

Other groups have explored whether LGALS3, in combination with other markers, could improve the diagnostic accuracy of FNA. It has been suggested a panel that includes LGALS3, FN1 and/or HBME1 [45]. Although the combination of these markers detects most carcinomas, false-positive results have been reported [46-48]. 

Comparative genome-wide transcriptional profiling has been widely used for identifying molecular markers. Using microarray analysis, Barden *et al.,* described a 105-gene expression classifier that differentiated between the FTA from FTC with high accuracy [49]. 

Based on the combination of *PCSK2*,* CCDN2* and *PLAB* expression, the authors developed a new model that predict the diagnosis of FTC and FTA with high sensitivity (100%) and specificity (96.7%) [50]. 

*TFF3* has been described as the most promising marker for diagnosis of FTC. The authors found that *TFF3* expression is markedly decreased in malignant thyroid carcinomas [51]. Further analysis demonstrated that TFF3 was highly expressed in normal thyroid compared to thyroid tumors, although no difference was reported among FTA, FTC, PTC and FVPTC by immunohistochemistry [52]. It has been suggested that the expression of *TFF3*, along with other molecular markers, may improve diagnosis of thyroid nodule [53,54].

## AN ANTIBODY-BASED TEST FOR THE DIAGNOSIS OF THYROID NODULE FNAS CLASSIFIED AS INDETERMINATE

We previously performed gene expression profiling of FTC, FTA and normal thyroid (http://cgap.nci.nih.gov/SAGE) [55]. This profiling and subsequent validation using quantitative PCR (qPCR) revealed that four genes (*DDIT3, ITM1*, *ARG2, C1orf24*) differed between FTA and FTC, and a linear combination of expression levels distinguish the benign from malignant with a predictive accuracy of 83% [56]. The four candidate markers were over-expressed in FTC and, therefore, carcinoma markers. The two commercially available antibodies (ARG2 and DDIT3) were validated in an independent set of FTA and FTC paraffin-embedded samples (*n*=59). We achieved a specificity of 90.6% and sensitivity of 85.2%. We next produced and tested antibodies for ITM1 and C1orf24 in the thyroid samples previously tested for DDIT3 and ARG2. In addition, we also tested all carcinoma markers on a wider range of benign and malignant lesions (*n*=127; FTA, HN, FTC, FVPTC and PTC). By adding C1orf24 and ITM1, we achieved a specificity of 90% and sensitivity of 100% [57]. However, the two best markers for detecting malignancy (C1orf24 and ITM1), had the drawback of few false-positives cases. All false-positive were benign lesions with Hürthle components. 

To improve the accuracy of our test, we next performed two SAGE libraries; one for the false-positive HCA and one for a HCC. Transcripts specifically expressed in HCAs were next validated by qPCR (*n*=76; FTA, HCA, FTC, CCH and FVPTC). To validate the results obtained from qPCR analysis and to define the final panel that would yield best diagnostic accuracy, the two best HCA markers (PVALB and KLK1) were tested, in combination with carcinoma markers (DDIT3, ITM1, ARG2 and C1orf24), in an independent set of 82 thyroid paraffin-embedded sections. We identified PVALB as a new marker that can be used in combination with C1orf24 and ITM1 for a more accurate diagnosis of thyroid nodules. By adding PVALB we achieved a specificity of 97% and maintained sensitivity for detection of carcinoma. 

Importantly, this antibody-based test was not only more accurate than mRNA based test but also these antibodies can effectively be used in FNA specimens [57,58]. 

Other groups have also investigated the expression of DDIT3, ITM1 and C1orf24 in thyroid tumors. C1orf24 expression was found in malignant thyroid tumors and in benign lesions scattered cells with Hürthle metaplasia [59]. 

A multigene marker panel that includes EMMPRIN, Autotaxin, DDIT3, LGALS3 and TFF3 was tested in FTC, FVPTC and FTA [60]. The authors reported a sensitivity of 90% for DDIT3, although they found a specificity of 21%. Given that our false-positive cases were HCA, one possible explanation for the decrease in specificity is that adenomas tested may have Hürthle cell components. This sample set also showed very low sensitivity (65% and 40%) and specificity (62% and 51%) for LGALS3 and TFF3, correspondingly*. *

Recently, a combination of *TFF3*, *STT3A* (*ITM1*) and *C1orf24* was tested in FNA biopsy [61]. In these set of samples, the expression of *TFF3* did not differ between benign and malignant. Although *ITM1* was differentially expressed between FTA and malignant tumors, it was detected in benign lesions. Similarly, the authors found *C1orf24* expression in few benign lesions. Whether the benign lesions tested have Hürthle cell components, it is not clear. However, we and others have previously reported that ITM1 and C1orf24 give false-positive results in HCA and benign lesions with Hürthle cells [56-59,62]. Additionally, the authors have investigated *ITM1* and *C1orf24* at mRNA levels and the expression of these markers performed in paraffin-embedded sections showed to be more robust than quantitative PCR performed in snap-frozen samples or FNA samples. 

All together, these findings suggests that a panel of markers that are able to distinguish all thyroid lesions may represent a helpful adjunct to the FNA cytology in order to rule in malignancy as a probable diagnosis, thus guiding the selection of patients who might benefit from thyroidectomy. Our findings suggest that o antibody-based test provide greatest cost effective potential for improving the diagnosis from FNA samples, further analysis testing these markers in a greater number of FNA specimens will clarify whether it can effectively help the preoperative evaluation of nodules classified as indeterminate and, therefore, be incorporated in most laboratories for the routine evaluation of thyroid nodules. 

## FUTURE PERSPECTIVES IN DIAGNOSIS OF THYROID NODULES

The PI3K pathway has been implicated in the pathogenesis of thyroid tumors [21,63-66]. Liu *et al.* indentified genetic alterations in genes along the PI3K/AKT pathways in FTC and undifferentiated thyroid carcinoma (UTC). Mutations and copy number gain were commonly found in UTC, although they were much less common in FTC. Copy gains of *PIK3CB *(45%) and mutation in *RAS* (20%) were the most common genetic alteration found in FTC. Although *PIK3CB* copy number gain was identify in a number of malignant lesions, this genetic variation should be further investigated in FTA samples [21].

We recently identified 6-non synonymous changes in *IDH1* gene in a panel of 164 thyroid samples [67]. The non synonymous mutations identified in our study were not identified in matched thyroid normal samples, suggesting that they were somatically acquired. Remarkably, *IDH1* mutations were highly prevalent in FTC, HCC and in FVPTC, while were not identified in the benign FTA and its variant [67]. Although these data indicate that IDH1 mutation analysis might help the diagnosis of indeterminate thyroid nodules, further analysis is still needed to determine the role of IDH1 in the pathogenesis of thyroid tumors and if its mutations preferentially accumulate in specific tumor subtypes. Interestingly, others have also indentified IDH1 variants in FTC and UTC [68]. 

In addition to the specific biomarkers found through gene expression profiling or mutational analysis, several published reports have also suggested the role of microRNAs (miRNAs) in the diagnosis of thyroid tumors. In fact, the genome-wide miRNA expression profile revealed that miR-197 and miR-346 are over-expressed in FTC compared to FTA [69]. Additionally miR-221 and miR-222 have been found to be consistently over-expressed in PTC [70-74]. It is still not clear whether the measurement of the miRNAs levels is a suitable tool for the preoperative diagnosis of thyroid nodules classified as indeterminate by FNA. However, some authors believe that miRNA will add significantly to the diagnosis of thyroid nodules [75]. One potential problem one might face is the small RNA fraction obtained from FNA biopsy, and, therefore, whether this approach is technologically feasible or not. Additionally, the difference observed in miRNA expression may not be sufficient to distinguish a benign from a malignant thyroid nodule. 

## THE NEXT STEPS

Currently, several studies have evaluated the diagnostic potential of candidate tumor markers (alone or in combination). Although very promising classifiers have been described, a common criticism is that the results are often contradictory, even for the most commonly used markers or for the panel of tumor-associated mutation. 

From the technical standpoint, one can predict that will happen for all markers discussed here. One possible explanation is that studies use diverse protocols, very few markers have been validated in multiple studies, the analyses is performed in different set and/or in small set of samples. 

Before the development of a preoperative diagnostic test that can be used in routine, we need to take into account several issues. Markers are usually validated on frozen sections or paraffin-embedded samples. FNA specimen may have contaminations with non-tumor cells. Therefore, assays based on quantitative level of expression (mRNA or protein) and mutation-based diagnostic test should take into account both tumor heterogeneity and contamination, all of which may reduce the sensitivity and specificity. Moreover, assays based on RNA are a challenge for routine clinical pathology, not only because RNA is much more susceptible to degradation than DNA but also because mRNA yields from FNA samples are variable and may not provide enough material for multigene analysis. Assays, which are observer-dependent, should be avoided. Therefore, a minimal number of genes are required and qualitative (positive or negative) results is desirable. 

Finally, a next important step for candidate molecular markers for thyroid tumors is the clinical testing of FNA samples in large multi-institutional trials. 

## Figures and Tables

**Table 1.** Diagnosis of Nodules Classified as Indeterminate on FNA Using a Panel of Mutations

Studies	Samples	Markers	Sensitivity (%)	Specificity (%)	PPV	NPV	Histology^A^	False-Negative	False-Positive
[24]	*n*=52	BRAF V600E NRAS (61) HRAS (61) KRAS (12, 13) RET/PTC 1 RET/PTC 3 PAX8/PPARγ	71 (100,75, 60)^B^	100 (100, 100,100)^B^	100 (100,100,100)^B^	83 (100,79,50)^B^	17 PTC, 4 FTC, 4 FTA and 27 HN	6/21 (4 PTC and 2 FTC)	0
[32]	*n*=41^C^	BRAF V600E NRAS (12, 13, 61) HRAS (12, 13, 61) KRAS (12, 13, 61) RET/PTC 1 RET/PTC 3 TRK PAX8/PPARγ	87	97	85	97	7 PTC, 26 FTA and 8 HN	1/7 (PTC)	1 FTA
[32]	*n*=54^D^	BRAF V600E NRAS (12, 13, 61) HRAS (12, 13, 61) KRAS (12, 13, 61) RET/PTC 1 RET/PTC 3 TRK PAX8/PPARγ	80	100	100	47	46 PTC, 4 FTA and 4 HN	9/46 (PTC)	0
[33]	*n*=110	BRAF V600E NRAS (12, 13, 61) KRAS (12, 13, 61) RET/PTC 1 RET/PTC 3 NTRK1	27 (12)^E^	95 (98)^E^	66 (38)^E^	78 (65)^E^	29 FTA, 6 HCA, 40 HN, 6 LT, 19 FVPTC, 8 FTC and 2 HCC	21 (4 FTC, 2 HCC, 15 FVPTC)	4 FTA
[34]	*n*=117	BRAF V600E NRAS (61) HRAS (61) KRAS (12, 13) RET/PTC 1 RET/PTC 3 PAX8/PPARγ	63	100	100	93	79 non-neoplastic, 18 FTA and 20 PTC,	8 PTC	0

A PTC, papillary thyroid carcinoma; FTC, follicular thyroid carcinoma; FTA, follicular thyroid adenoma; HN, hyperplasia; HCA, Hürthle cell adenoma; HCC, Hürthle cell carcinoma; FVPTC, follicular variant of PTC; LT, lymphocytic thyroiditis.

B When indeterminate nodules were assigned to the propose NCI categories (FLUS, follicular neoplasm or suspicious for malignancy) the sensitivity was 100%, 75% and 60%, respectively. The PPV and NPV are given according to NCI categories.

C Nodules classified as indeterminate.

D Nodules classified as suspicious for malignancy.

E Results including nodules classified as indeterminate (*n*=110) and suspicious for malignancy (*n*=27). All false-positive were due to *RAS* mutation.
